# Numerical Analysis of Highly Sensitive Twin-Core, Gold-Coated, D-Shaped Photonic Crystal Fiber Based on Surface Plasmon Resonance Sensor

**DOI:** 10.3390/s23115029

**Published:** 2023-05-24

**Authors:** Md. Ranju Sardar, Mohammad Faisal

**Affiliations:** Department of Electrical and Electronic Engineering, Bangladesh University of Engineering and Technology, Dhaka 1205, Bangladesh; mdfaisal@eee.buet.ac.bd

**Keywords:** photonic crystal fiber, surface plasmon resonance, refractive index, finite element method, COMSOL Multiphysics

## Abstract

This research article proposes and numerically investigates a photonic crystal fiber (PCF) based on a surface plasmon resonance (SPR) sensor for the detecting refractive index (RI) of unknown analytes. The plasmonic material (gold) layer is placed outside of the PCF by removing two air holes from the main structure, and a D-shaped PCF-SPR sensor is formed. The purpose of using a plasmonic material (gold) layer in a PCF structure is to introduce an SPR phenomenon. The structure of the PCF is likely enclosed by the analyte to be detected, and an external sensing system is used to measure changes in the SPR signal. Moreover, a perfectly matched layer (PML) is also placed outside of the PCF to absorb unwanted light signals towards the surface. The numerical investigation of all guiding properties of the PCF-SPR sensor is completed using a fully vectorial-based finite element method (FEM) to achieve the finest sensing performance. The design of the PCF-SPR sensor is completed using COMSOL Multiphysics software, version 1.4.50. According to the simulation results, the proposed PCF-SPR sensor has a maximum wavelength sensitivity of 9000 nm/RIU, an amplitude sensitivity of 3746 RIU^−1^, a sensor resolution of 1 × 10^−5^ RIU, and a figure of merit (FOM) of 900 RIU^−1^ in the x-polarized direction light signal. The miniaturized structure and high sensitivity of the proposed PCF-SPR sensor make it a promising candidate for detecting RI of analytes ranging from 1.28 to 1.42.

## 1. Introduction and Literature Review

In recent decades, photonic crystal fiber (PCF)-based surface plasmon resonance (SPR) sensors [1,2,3,4,5,6,7] have been attractive to many sensor researchers due to their outstanding features, which include controllable dispersion and birefringence, low loss, high sensitivity, good sensor resolution, fast response time, label-free detection, real-time monitoring, reliability, operational flexibility, simple and miniaturized structure, and tunable structural parameters [8,9,10,11,12,13]. PCF-SPR sensors have shown potential for use in various fields, including bimolecular analyte detection, medical diagnosis, medical testing, blood group detection, virus detection, cancerous cell detection in the human body, drug testing, food quality control for safety, environmental monitoring, and other applications [14,15,16,17,18]. An SPR is the collective oscillation of free electrons at metal–dielectric interfaces that are stimulated by the photons of an evanescent optical field [19,20,21]. It occurs when the wavelength of free electrons and the wavelength of photons are in resonance with each other at the metal–dielectric interface [22,23]. However, the plasmonic effect refers to the interaction between the free electrons and the photons of evanescent optical field at the metal–dielectric interface [24,25,26,27]. The core of PCF has a higher refractive index than the cladding, which allows a small amount of light to penetrate into the cladding region [28]. This light interacts with the plasmonic material layer at the metal–dielectric interface, exciting the free electrons and creating a surface plasmon wave (SPW) [29]. SPWs show the highest intensity in metal–dielectric crossing points, and they deplete gradually with the depth of the dielectric layer [30]. When the wavelength of the evanescent field matches the wavelength of the SPW, a phenomenon called SPR occurs [31]. This is also known as the resonance condition or phase-matching condition, and the wavelength at which it occurs is referred to as the resonance wavelength [32]. There are instances in which a peak in the resonance curve occurs due to the transfer of the highest photon energy from the core mode to the surface plasmon polariton (SPP) mode [33]. This peak is unique to each analyte and is used to detect the presence and concentration of the analyte [34]. Therefore, by measuring the resonance peak loss curve, one can determine the specific analyte present and its concentration. There are three basic types of surface plasmon resonance (SPR) sensors: prism-based [35], fiber grating-based [36], and photonic crystal fiber (PCF)-based sensors [37]. Prism-based SPR sensors are bulky, and their components include a dielectric layer, metal film substrate, liquid, and plasmonic material [35]. Fiber-grating-based SPR sensors are an intermediate option that provides lower sensitivity and a broader peak loss curve, making it difficult to identify unknown analytes [36]. PCF-SPR sensors offer a sharp resonance peak loss curve, good sensing performance, and ease of practical fabrication [37]. In contrast, the PCF-SPR sensor is smaller and easier to fabricate than others [38,39]. The sensing performance of a PCF-SPR sensor depends on the choice of background material and plasmonic material [40,41]. Fused silica is a preferred background material because it is cost-effective and does not require complex fabrication processes [42]. The most well-known plasmonic materials are copper (Cu), silver (Ag) and gold (Au) [43,44,45,46]. Among the commonly used plasmonic materials, gold (Au) is known for its chemical stability and resistance to oxidation in adverse environments [43]. This makes it a preferred material for plasmonic sensors, as it can provide long-term service with consistent and reliable sensing performance [43]. In contrast, materials like copper (Cu), and silver (Ag) are not chemically stable and can easily be oxidized, leading to degradation of sensing performance over time [44,45,46]. While coating these materials with a layer of graphene can prevent oxidation and improve their stability, it also adds complexity to the fabrication process [47]. Therefore, gold remains a popular choice for plasmonic sensors due to its stability and durability [48]. Two approaches to SPR sensing systems are available: internal sensing [49] and external sensing [50]. Internal sensing systems involve coating plasmonic material on the external wall of selected air holes inside the design and filling analyte into these air holes [51]. However, this approach faces several challenges, including high propagation loss, complexity in filling analyte into small air holes, and fabrication complexity in creating identical metal coatings on the external wall of small air holes [52]. In contrast, external sensing systems offer several advantages over internal sensing systems. In this approach, an analyte layer is placed outside the photonic crystal fiber (PCF) structure, and analytes can be easily circulated through it by applying a programmable pump [50]. This approach eliminates the challenges faced by internal sensing systems, such as filling analytes into small air holes and fabrication complexity [52]. Several researchers have proposed PCF-SPR sensors with an external sensing system. For instance, Dash et al. proposed a silver-graphene coated D-shaped PCF-SPR sensor for detecting the RI of analytes. Authors achieved an amplitude sensitivity of 216 RIU^−1^, a wavelength sensitivity of 3700 nm/RIU, and a sensor resolution of 2.7 × 10^−5^ RIU^−1^ [53]. However, the achievable values of amplitude sensitivity and wavelength sensitivity were found to be insufficient, and the authors did not consider the figure of merit (FOM). Rifat et al. proposed a PCF-SPR sensor with a honeycomb structure for detecting the RI of analytes [54]. They used a graphene layer coating on top of the plasmonic material layer to prevent oxidation and enhance sensing performance [54]. However, the use of the graphene layer coating increased the manufacturing cost, sensor size, and fabrication complications [47]. Wang et al. reported a highly sensitive D-shaped PCF-SPR sensor for detecting RI values from 1.345 to 1.41 [55]. Although authors achieved a high wavelength sensitivity of 12,450 nm/RIU, the RI range of analytes was very small, and they did not calculate the amplitude sensitivity, sensor resolution, or FOM [55]. Finally, Wu et al. suggested a D-shaped PCF-SPR sensor for detecting RI values from 1.32 to 1.40 and achieved a high wavelength sensitivity of 31,000 nm/RIU [56]. They also did not calculate the amplitude sensitivity, sensor resolution, or FOM [56]. In summary, while several PCF-SPR sensors with external sensing systems have been proposed, each has its advantages and limitations. Future research should focus on developing sensors with improved sensitivity and FOM while keeping the manufacturing cost, sensor size, and fabrication complexity low. The paper proposes a twin-core, gold-coated, D-shaped PCF-SPR sensor that overcomes the limitations of previously reported sensors by determining four sensing parameters: wavelength sensitivity, amplitude sensitivity, sensor resolution, and figure of merit (FOM). The D-shaped PCF-SPR sensor proves to be a more economical option in comparison to circular-shaped PCF-SPR sensors due to its utilization of a smaller amount of plasmonic material and a reduced distance between the plasmonic layer and the core. These features result in a higher intensity of the evanescent field on the plasmonic material layer, allowing for more oscillations of free electrons in metal–dielectric interfaces and improved overall sensing performance.

## 2. Block Diagram and Mode of Operation

**Figure 1:**
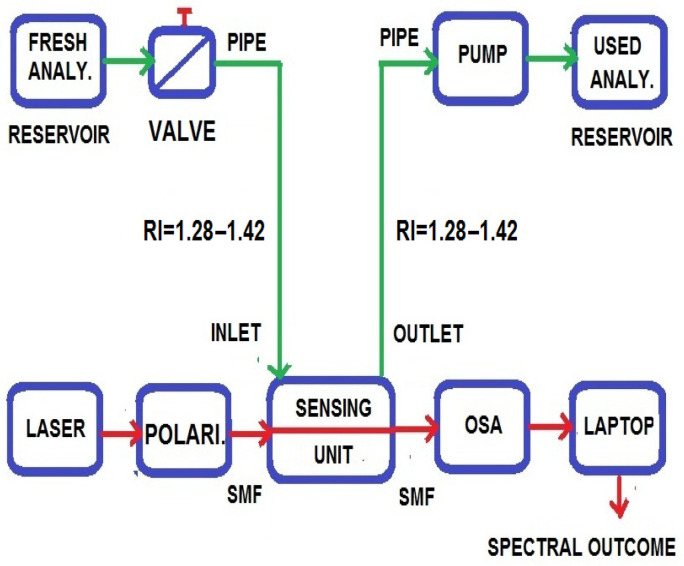
Basic block diagram of D-shaped PCF-SPR sensor.

Figure 1 shows the experimental setup and operating principle of the proposed sensor. The setup includes various components, such as light amplification by stimulated emission of radiation (laser), an optical polarizer, a sensing unit, an optical spectrum analyzer (OSA), a laptop, a programmable pump, an analyte reservoir, and more. The laser light is coupled into the optical polarizer unit using an SMF fiber, which converts unpolarized light into polarized light and passes it through the sensing unit. Analyte is also introduced into the sensing unit using the PVC pipe, and the valve controls the flow of analyte with a standard pressure and temperature. The programmable pump circulates the analytes into the sensing unit step by step and finally discharges them into the waste reservoir. The interaction between the free electrons of plasmonic material and photons of the evanescent optical field takes place in the sensing unit. The OSA receives the polarized light signal from the sensing unit using SMF and quantifies the optical spectra. The laptop gathers the quantified optical spectra from OSA using SMF and displays the resonance peak loss curve as a monitor corresponding to each unknown analyte with a distinct resonance wavelength. By analyzing the resonance peak loss curve, the sensor is capable of detecting and identifying different analytes with high precision.

## 3. Sensor Design and Optimization Process

**Figure 2:**
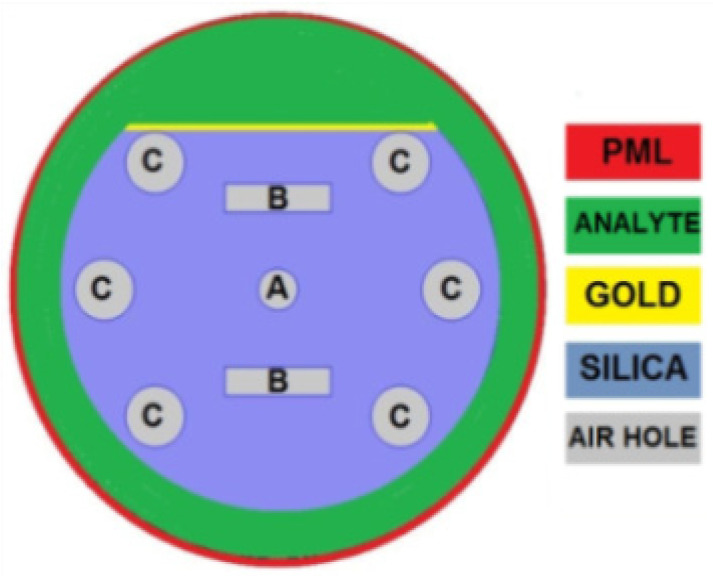
Cross-sectional view with different parameters of a designed PCF-SPR sensor.

Figure 2 shows a cross-sectional view of the D-shaped PCF-SPR sensor, which consists of a central air hole, two air hole rings, a gold layer, an analyte layer, and a PML layer. The first and second rings are made up of rectangular and circular air holes, respectively. The central air hole (A) is located in the core, while air holes B and C are located in the cladding to create a difference in RI between the core and cladding. The gold layer is positioned outside of the PCF structure, and two air holes are removed to create a D-shaped sensor. The analyte layer is also located outside the PCF structure, and analyte is circulated through it using a programmable pump. The PML layer is designed to improve sensing performance by absorbing unwanted light signals and canceling the reflection of light. All structural parameters were optimized step-by-step to achieve the best sensing performance. The optimum areas were found to be 0.0314 µm^2^, 0.6359 µm^2^, and 1.0 µm^2^ for air holes A, B, and C, respectively. Correspondingly, the optimum thicknesses were found to be 26 nm, 580 nm, and 80 nm for the gold layer, analyte layer, and PML, respectively.

## 4. Resonance Conditions and Optimum Energy Transfers System

**Figure 3:**
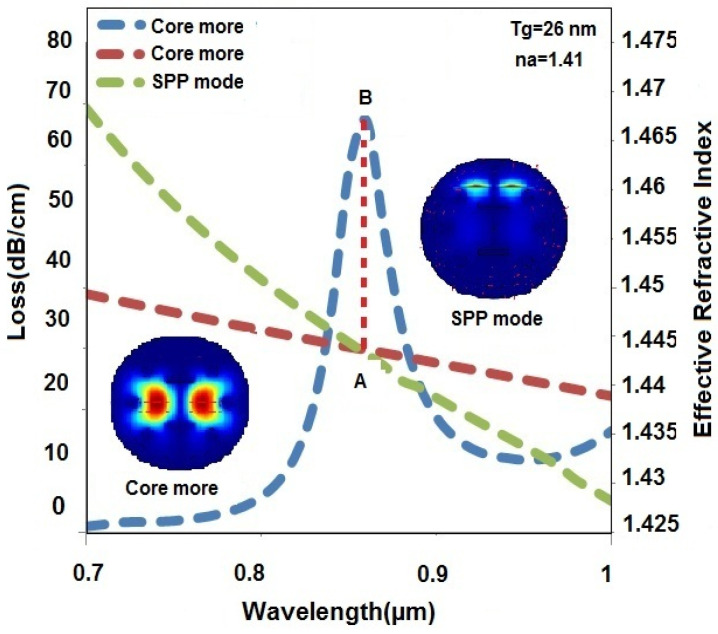
Core mode and SPP mode of a designed PCF-SPR sensor as well as their relationship for a specific scenario involving an x-polarized direction light signal with an analyte RI of 1.41 and a gold layer thickness of 26 nm.

Figure 3 shows the maximum photon energy transfers from the core mode to the surface plasmon polariton (SPP) mode at resonance condition or phase matching condition. The blue and brick-colored dashed lines in Figure 3 characterize the loss and real part of the effective RI of the core mode, respectively. Furthermore, the green dashed line characterizes the effective RI of the SPP mode. From Figure 3, it can be observed that the loss of the core mode increases with increasing wavelength up to the resonance point (B) and later starts decreasing with increasing wavelength. On the other hand, the real part of effective RIs of the core mode and SPP mode decrease with increasing wavelength. Additionally, the real part of effective RI of the core mode suitable matches the effective RI of SPP mode and satisfies the resonance condition. In Figure 3, the resonance occurs at λ = 0.86 µm wavelength, and maximum photon energy transfers from the core mode to the SPP mode instantaneously. This resonance condition is crucial for the operation of the proposed sensor because it enables efficient and accurate detection of various analytes based on the interaction between the plasmonic material and the photons of the evanescent optical field.

## 5. Analysis of Mathematical Equations

A small amount of light signal transfers from the core mode to SPP mode in a PCF due to the difference in RI between the core and cladding. This phenomenon is translated as losses affecting the light. Loss is typically quantified in terms of the imaginary part of the effective RI of the modes involved. The loss profile of the PCF-SPR sensor can be stated using the following equation [57]:

(1) $$\alpha \left(\frac{dB}{cm}\right)=8.686\times K_{0}I_{m}\left[n_{eff}\right]\times 10^{4}$$

where $K_{0}=\frac{2\pi }{\lambda }$ represents the wave number in free space, which is equal to 2*π* divided by the wavelength (*λ*) of the light. The $I_{m}\left[n_{eff}\right]$ represents the effective RI that is related to the attenuation or loss of the light signal as it propagates through PCF. The wavelength sensitivity is the ratio of the change in resonance wavelength ${(\Delta \lambda }_{peak})$ to the change in RI of an adjacent analyte. It is a measure of how much the resonance wavelength shifts in response to a change in the surrounding RI. Typically, the resonance wavelength shifts towards a higher value as the RI increases. Wavelength sensitivity can be calculated using the wavelength interrogation method, which can be quantified using the following equation [58]:

(2) $$S_{\lambda }(\frac{nm}{RIU})=\left(\frac{{∆\lambda }_{peak}}{{∆n}_{a}}\right)$$

where ${\Delta \lambda }_{peak}$ refers to the difference in wavelength between the peak positions of two spectral peaks and ${\Delta n}_{a}$ refers to the effective RI difference between two adjacent analytes. The sensor resolution is a key performance parameter of a PCF-SPR sensor, which represents the ability of the sensor to resolve small changes in the RI of the analyte. The resolution of the PCF-SPR sensor can be defined as the minimum detectable change in the RI that can be calculated using the following equation [59]:

(3) $$R=\frac{{∆n}_{a}{∆\lambda }_{min}}{{∆\lambda }_{Peak}}$$

where R denotes the smallest detectable change in the sensor output, ${\Delta n}_{a}$ denotes the difference in RI between two adjacent analytes, ${\Delta \lambda }_{peak}$ denotes the difference in wavelength between the resonance peaks of the sensor for two adjacent analytes, and ${\Delta \lambda }_{min}$ is a constant value of 0.1 nm that is used in OSA to set the minimum detectable wavelength resolution. The amplitude sensitivity is a considerable sensing parameter of the sensor, which refers to the change in the amplitude of the output signal due to changes in the RI of the analyte. Amplitude sensitivity can be calculated using the amplitude integration method, which can be defined using the following equation [60]:

(4) $$S_{A}=-\frac{1}{\alpha (\lambda ,n_{a})}\frac{\delta \alpha (\lambda ,n_{a})}{{\delta n}_{a}}$$

where $S_{A}$ describes the change in the amplitude of the sensor signal per unit change in RI and $\delta_{a}$(${\lambda ,n}_{a})$ describes the loss difference between two analytes, usually expressed in units of dB/cm. Wavelength sensitivity and full-width half maximum (*FWHM*) are two important parameters that are often used to analyze the performance of PCF-SPR sensors. However, the *FOM* is a term that is often used to characterize the overall performance of a PCF-SPR sensor. The sharp resonance curve and the utmost wavelength sensitivity collectively generate maximum *FOM*, which can be expressed using the following equation [61]:

(5) $$FOM=\frac{S_{\lambda }}{FWHM}$$

The Sellmeier equation is an important mathematical tool used to calculate the RI of a material. However, the Sellmeier equation is commonly used in the design and optimization of PCF-SPR sensors. Mathematically, the Sellmeier equation with its different parameters can be expressed as [62]:

(6) $$n^{2}(\lambda )=1+\frac{B_{1}\lambda^{2}}{\lambda^{2}-C_{1}}+\frac{B_{2}\lambda^{2}}{\lambda^{2}-C_{2}}+\frac{B_{3}\lambda^{2}}{\lambda^{2}-C_{3}}$$

where *n* is the effective RI of the fused silica at a given operating wavelength *λ*, whereas $B_{1},$ $B_{2},B_{3}$ and $C_{1},C_{2},C_{3}$ are the Sellmeier constants for fused silica; their numerical values are approximately 0.69616300, 0.407942600, 0.407942600, 0.897479400, 0.00467914826, 0.0135120631, and 97.9340025, respectively. The Drude–Lorentz model is utilized to calculate the permittivity of gold, which is a measure of how a material responds to electric fields. Mathematically, the Drude–Lorentz model can be expressed using the following equation [63]:

(7) $${ɛ}_{AU}={ɛ}_{\infty }-\frac{{\omega^{2}}_{D}}{\omega (\omega +jY_{D})}-\frac{∆ɛ{{Ω}^{2}}_{L}}{{(\omega }^{2}-{{Ω}^{2}}_{L})+jT_{L}\omega }$$

where ${ɛ}_{AU}$ characterizes the permittivity of gold, ${ɛ}_{\infty }$ also characterizes the permittivity of gold at the utmost frequency with a value of 5.9673, *ω* characterizes the angular frequency that is given by *ω* = 2*πc*/*λ*, c is the velocity of light in the medium,$\omega_{D}$ characterizes the plasma frequency, $Y_{D}$ characterizes the damping frequency, ∆ɛ characterizes the weighting factor, $T_{L}$ characterizes the spectral width, and ${Ω}_{L}$ quantifies the oscillator strength. Moreover, different constants of Lorentz oscillator are given as $\omega_{D}$ = 13,273.408 THz, $Y_{D}$ = 100 THz, ∆ɛ = 1.09, $T_{L}$= 658.53 THz, and ${Ω}_{L}$= 650.07 THz, respectively.

The sensor length of a PCF-SPR sensor refers to the length of the PCF used in the sensing element. In general, the sensor length is inversely proportional to the loss, which can be stated using the following equation [48]:

(8) $$L=\frac{1}{\alpha (\lambda ,n_{a})}$$

where *L* indicates the length of sensor and $\alpha (\lambda ,n_{a})$ indicates the loss of PCF-SPR sensor.

## 6. Results and Performance Analysis

### 6.1. Loss and Amplitude Sensitivity Change with RI of Analyte

**Figure 4:**
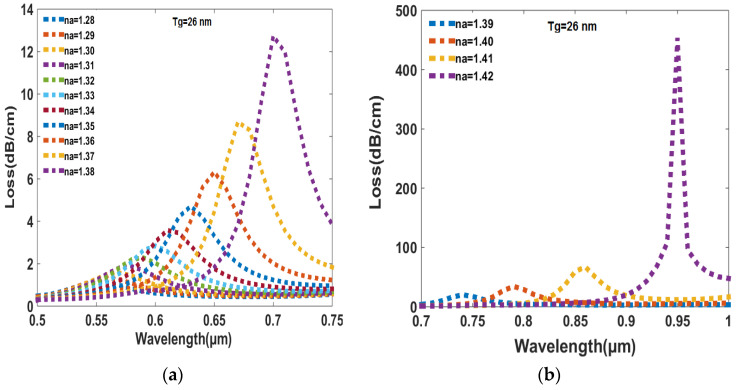
(**a**)The peak loss varies as a function of wavelength for the RI of analytes ranging from 1.28 to 1.38. (**b**)The peak loss is affected by changes in wavelength for the RI of analytes ranging from 1.39 to 1.42.

In Figure 4a,b, the peak amplitude and resonance wavelength are shown as functions of the RI of the analyte. It can be observed that as the RI of the analyte increases, the peak loss and resonance wavelength shift towards higher wavelengths. This is because a higher RI of the analyte allows for more efficient transfer of photon energy from the core mode to the SPP mode, leading to a stronger interaction between the plasmonic material and the analyte. In Figure 4a, the lowest RI of 1.28 provides a peak loss and resonance wavelength of 1.1256 dB/cm and 0.56 µm, respectively. On the other hand, in Figure 4b, the highest analyte RI of 1.42 provides a peak loss and resonance wavelength of 775.7314 dB/cm and 0.95 µm, respectively. This indicates that the peak loss and resonance wavelength increase with increasing RI of the analyte.

**Figure 5:**
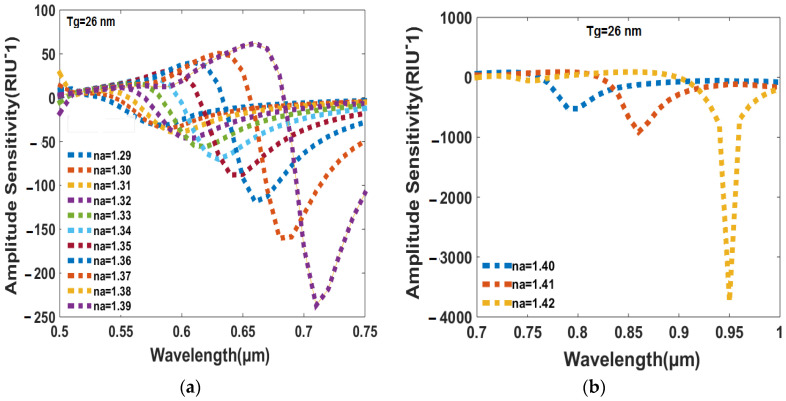
(**a**) The amplitude sensitivity varies with wavelength across the RI range of analytes from 1.29 to 1.39. (**b**) The wavelength-dependent variation of amplitude sensitivity occurs within the RI range of analytes from 1.40 to 1.42.

Figure 5a shows the relationship between the amplitude sensitivity and the RI of the analyte in the range of wavelengths from 0.5 µm to 0.75 µm, while Figure 5b shows the same relationship in the range of wavelengths from 0.7 µm to 1 µm. In Figure 5a, it is observed that the amplitude sensitivity decreases as the RI of the analyte decreases. The lowest analyte RI of 1.29 offers the minimum amplitude sensitivity of −31.3674 RIU^−1^, while the highest analyte RI of 1.38 offers the maximum amplitude sensitivity of fe−236.8681 RIU^−1^. In Figure 5b, a similar trend is introduced in which the minimum amplitude sensitivity is obtained for the lowest RI of 1.40, and the maximum amplitude sensitivity is obtained for the highest RI of 1.42. However, the magnitude of the amplitude sensitivity is much higher compared to Figure 5a, with the maximum amplitude sensitivity being −3746 RIU^−1^. Overall realization: it suggests that the sensing system is more sensitive to change in the RI of higher-index analytes at the specific wavelength. When the RI of the analyte is higher than 1.42, there is no light signal detected in either the x- or y-polarized directions in the core area of the sensor. This implies that the sensor is not able to detect the presence of the analyte at this RI range. Conversely, when the RI of the analyte is lower than 1.27, the resonance wavelength value of the analyte matches to the RI of 1.28, which means that the suggested design of the sensor is capable of efficiently detecting RI values in the range of 1.28 to 1.42.

### 6.2. Effect of Gold Layer Thickness (GLT) on Loss and Amplitude Sensitivity

**Figure 6:**
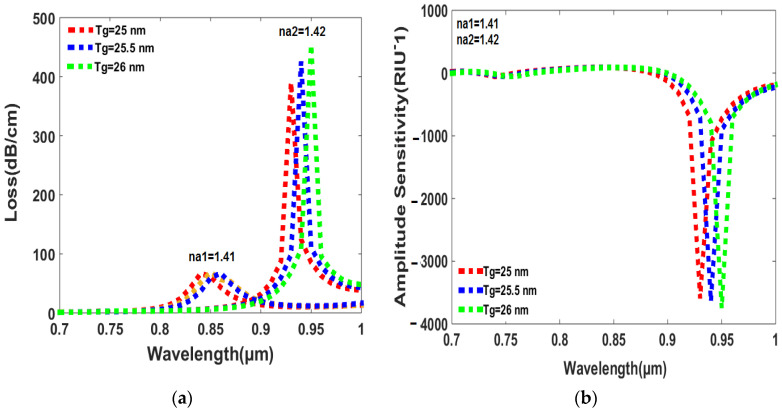
(**a**) The peak loss is a function of the gold layer thickness for the RI range of the analytes tested from 1.41 to 1.42. (**b**) The amplitude sensitivity varies as a function of the gold layer thickness within the RI range of analytes from 1.41 to 1.42.

Figure 6a shows that the peak loss varies with gold layer thickness and wavelength for an analyte RI of 1.41. For an RI of 1.41, the peak losses are 66.54 dB/cm, 68.73 dB/cm, and 67.34 dB/cm for gold layer thicknesses of 25 nm, 25.5 nm, and 26 nm, respectively. On the other hand, for an RI of 1.42, the peak losses are 400.08 dB/cm, 425.8 dB/cm, and 453.61 dB/cm for the same gold layer thicknesses, respectively. The highest RI of 1.42 offers the maximum peak loss compared to the lowest RI of 1.41, which is expected because maximum photon energy penetrates from the core to SPP modes at the higher RI value. The resonance wavelength also shifts towards higher wavelengths with increasing gold layer thickness and RI of analyte due to maximum damping loss happening at the highest gold layer thickness. The study finds that the optimum gold layer thickness lies between 25 nm and 26 nm. Thicknesses less than 25 nm or greater than 26 nm do not produce any x-polarized direction or y-polarized direction light signals in the core area, which is necessary to obtain true data. In Figure 6b, the three thicknesses are examined; the 26 nm gold layer thickness provides the highest amplitude sensitivity of 3746 RIU^−1^, while the sensitivities for 25 nm and 25.5 nm thicknesses are 3680 RIU^−1^ and 3710 RIU^−1^, respectively. Based on these findings, the study concludes that a 26 nm gold layer thickness is the optimum value for this design.

### 6.3. Effect of Central Air Hole Area on Loss and Amplitude Sensitivity

**Figure 7:**
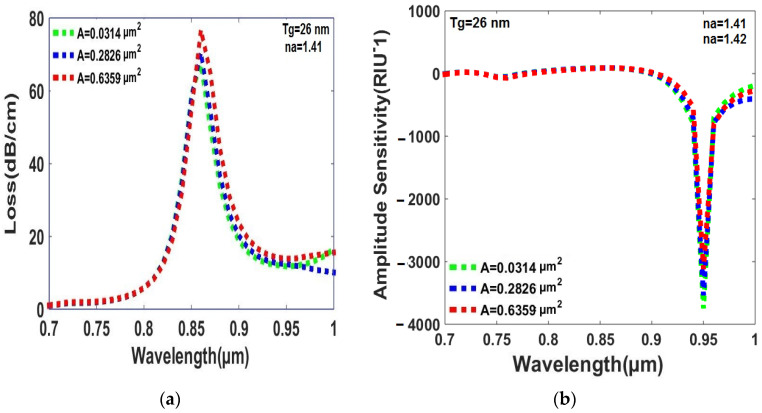
(**a**) The peak loss varies with changes in the area of the central air hole within the RI range of the analytes, which falls between 1.41 and 1.42. (**b**) The amplitude sensitivity is dependent on variations in the area of the central air hole over the RI range of the analytes, which spans from 1.41 to 1.42.

Based on the simulation results reported in Figure 7a,b, it can be concluded that the peak loss and amplitude sensitivity of the sensor are affected by the central air hole area. Specifically, increasing the air hole area leads to higher peak loss, while decreasing the air hole area leads to lower peak loss and higher amplitude sensitivity. In this study, the air hole area of 0.0314 µm^2^ is considered the best value for achieving superb sensing performance as it provides the highest amplitude sensitivity and the lowest peak loss. Overall, these findings suggest that careful optimization of the sensor design, including the central air hole area, is crucial for achieving optimal sensing performance.

### 6.4. Effect of Rectangular Air Hole Area of Cladding on Loss and Amplitude Sensitivity

**Figure 8:**
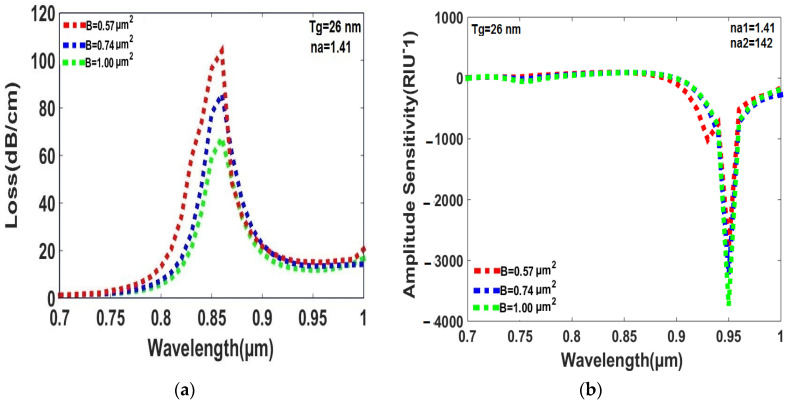
(**a**)The peak loss is affected by the rectangular air hole area of cladding for the RI range of the analytes, which is from 1.41 to 1.42. (**b**) The amplitude sensitivity changes with the area of rectangular air holes in the cladding area, over a RI range of analytes from 1.41 to 1.42.

According to the simulation results presented in Figure 8a,b, it can be observed that the peak loss and amplitude sensitivity vary with the area of the rectangular air holes for the analytes with RI values ranging from 1.41 to 1.42. In Figure 8a, the peak losses were found to be 79 dB/cm, 77.77 dB/cm, and 67.34 dB/cm for air hole areas of 0.57 µm^2^, 0.74 µm^2^, and 1.00 µm^2^, respectively. Where, the largest air hole area of 1.00 µm^2^ exhibits the smallest peak loss. Therefore, it is considered the optimal value for effectively detecting the RI of analytes with values ranging from 1.41 to 1.42. Moreover, the amplitude sensitivity values were found to be 2805 RIU^−1^, 3229 RIU^−1^, and 3746 RIU^−1^ for air hole areas of 0.57 µm^2^, 0.74 µm^2^, and 1.00 µm^2^, respectively. As observed in Figure 8b, the largest air hole area of 1.00 µm^2^ displays the highest amplitude sensitivity compared to the other two areas. Thus, it can be concluded that an air hole area of 1.00 µm^2^ is the optimal value for both the loss and amplitude sensitivity of the designed sensor.

### 6.5. Effect of Circular Air Hole Area of Cladding on Loss and Amplitude Sensitivity

**Figure 9:**
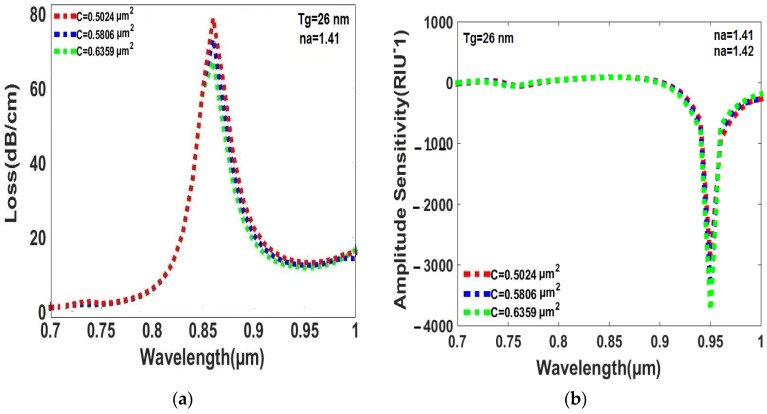
(**a**) The circular air hole area cladding has an impact on the peak loss for the analytes RI range, from 1.41 to 1.42. (**b**) The amplitude sensitivity is influenced by the circular air hole area in the cladding area, within the RI range of analytes from 1.41 to 1.42.

The peak loss and amplitude sensitivity are dominated by the circular air hole area of the cladding for the RI range of the analyte from 1.41 to 1.42, and the thickness of the gold layer is 26 nm. The peak loss and amplitude sensitivity of the sensor vary with the circular air hole area of the cladding as presented in Figure 9a,b. In Figure 9a, the maximum air hole area of 0.6359 µm^2^ displays the minimum peak loss, which is considered the best value for detecting the RI of analyte. In Figure 9b, a similar trend is found in which the maximum air hole area of 0.6359 µm^2^ provides the maximum amplitude sensitivity, with a value of 3746 RIU^−1^, compared to the minimum areas of 2943 RIU^−1^ and 3350 RIU^−1^ for air hole areas of 0.5024 µm^2^ and 0.5806 µm^2^, respectively. Therefore, the maximum air hole area of 0.6359 µm^2^ is selected as the optimal value for both peak loss and amplitude sensitivity.

### 6.6. Effect of PML Thickness on Loss and Amplitude Sensitivity

**Figure 10:**
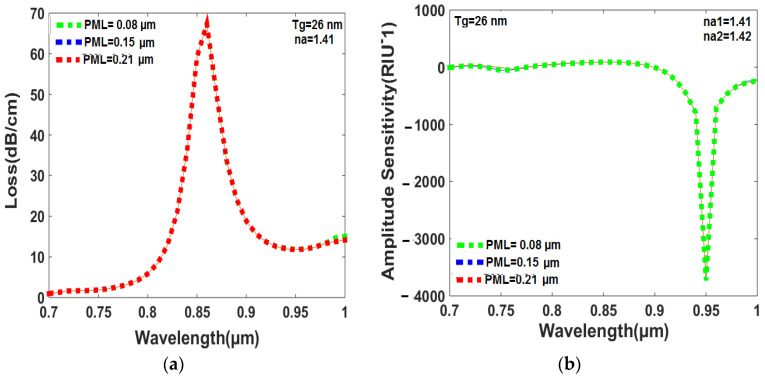
(**a**) The thickness of the PML impacts the peak loss within a RI range of analytes that falls between 1.41 and 1.42. (**b**) The amplitude sensitivity within a RI range of analytes between 1.41 and 1.42 is influenced by the thickness of the PML.

The peak loss and amplitude sensitivity change with the perfectly matched layer (PML) thickness, as displayed in Figure 10a,b. Figure 10a shows that the minimum PML thickness of 0.08 µm offers the lowest peak loss of 67.3408 dB/cm for the RI of 1.41, and the resonance wavelength does not shift towards higher wavelengths with increasing PML thickness. This is because a small amount of damping loss occurs with increasing PML thickness, which keeps the resonance wavelength in a fixed position. Figure 10b shows that the lower PML thickness of 0.08 µm provides the highest amplitude sensitivity of 3746 RIU^−1^ for the RI range of 1.41 to 1.42. The suggested structure is expected to display good sensing performance with a minimum PML thickness of 0.08 µm. It is noted that both peak loss and amplitude sensitivity slightly change with the PML thicknesses. However, the legend colors (red, blue, green) in Figure 10a,b are not able to display the loss and amplitude sensitivity results separately.

### 6.7. Effect of Analyte Layer Thickness (ALT) on Loss and Amplitude Sensitivity

**Figure 11:**
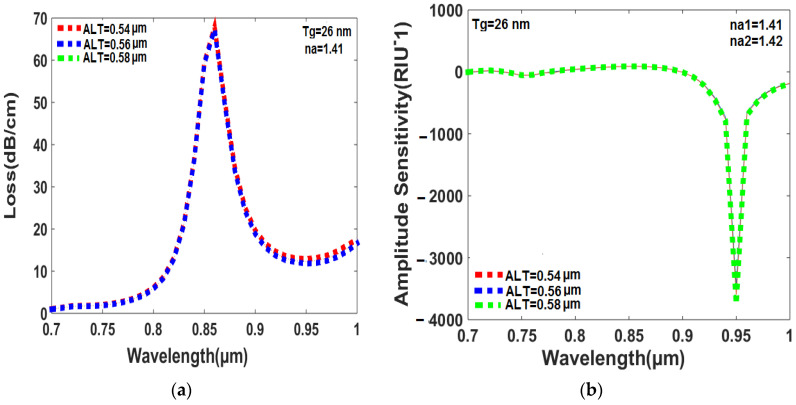
(**a**) The thickness of the analyte layer influences the peak loss when the RI of the analytes falls between 1.41 and 1.42. (**b**) The thickness of the analyte layer have an impact on the amplitude sensitivity with the RI of analytes ranging from 1.41 to 1.42.

The peak loss is regulated by the thickness of the analyte layer as shown in Figure 11a. In Figure 11a, the peak losses are found to be 67.8950 dB/cm, 67.6150 dB/cm, and 67.3408 dB/cm for analyte layer thicknesses of 0.54 µm, 0.56 µm, and 0.58 µm, respectively. The highest analyte thickness of 0.58 µm provides the least peak loss of 67.3408 dB, and the resonance wavelength does not shift towards the higher wavelength with increasing analyte layer thickness. This is because a small damping loss occurs with increasing analyte layer thickness, which stabilizes the resonance wavelength in a fixed position. In Figure 11b, a similar trend is realized in which the amplitude sensitivity of the sensor is affected by the thicknesses of the analyte layer. In particular, the amplitude sensitivity is found to increase with increasing analyte layer thickness, as denoted by the values of 3457 RIU^−1^, 3596 RIU^−1^, and 3746 RIU^−1^ for analyte layer thicknesses of 0.54 µm, 0.56 µm, and 0.58 µm, respectively. Moreover, when the analyte layer thickness is less than 0.54 µm or greater than 0.58 µm, the sensor cannot detect x-polarized direction and y-polarized direction light signals in the core area, which are necessary to calculate sensing parameters. The peak loss and amplitude sensitivity vary slightly with changing analyte layer thickness. Therefore, Figure 11a,b cannot display peak loss and amplitude sensitivity outcomes clearly according to their respective colors.

Table 1 presents a comparison of the sensing parameters of the proposed PCF-SPR sensor with those of previously published articles. Table 1 shows that some of the prior articles did not compute certain sensing parameters, such as sensor resolution, FOM, and amplitude sensitivity. In comparison, the designed sensor offers a good enough RI range of analytes, FOM, and amplitude sensitivity, suggesting that its sensing performance is better than that of the prior published articles.

Table 2 displays the optimal values of various geometrical parameters for the PCF-SPR sensor, which include the air holes areas of A, B, and C, the gold layer thickness (GLT), the analyte layer thickness (ALT), and the perfectly matched layer (PML). The optimal areas determined for air holes of A, B, and C were 0.0314 µm^2^, 0.6359 µm^2^, and 1.0 µm^2^, respectively. Additionally, the optimal thicknesses for the gold layer, analyte layer, and PML were determined as 26 nm, 580 nm, and 80 nm, respectively.

## 7. Correlation between Resonance Wavelength and Refractive Index (RI) of Analyte

**Figure 12:**
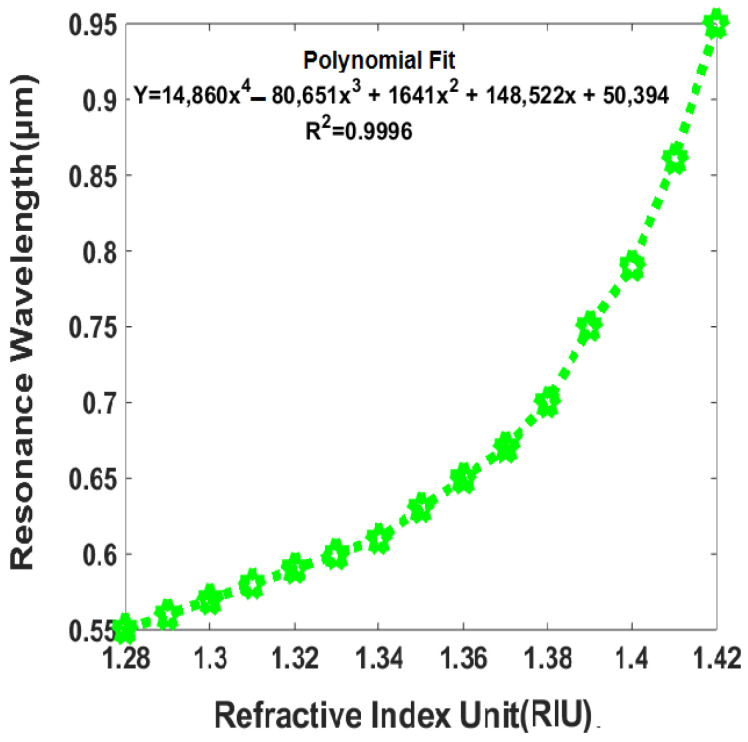
A polynomial fitting curve that illustrates how the resonance wave varies with respect to change in the RI of the analyte, specifically ranging from 1.28 to 1.42.

The polynomial fitting curve presented in Figure 12 provides a useful tool for predicting the resonance wavelength of the PCF-SPR sensor in response to changes in the RI of analytes. The formula of polynomial curve fitting states as Y = 14,680x^4^ − 80,650x^3^ + 1641x^2^ + 148,522x + 50,394, where Y represents the resonance wavelength and x represents the RI of the analyte. The researchers can estimate the expected resonance wavelength for a given change in RI using the formula: The high $R^{2}$ squared value of 0.9996 indicates that the polynomial fitting curve provides an accurate representation of the relationship between resonance wavelength and RI. This information is crucial for designing and optimizing sensors for particular applications.

## 8. Exploring the Correlation between Sensor Length and Loss

**Figure 13:**
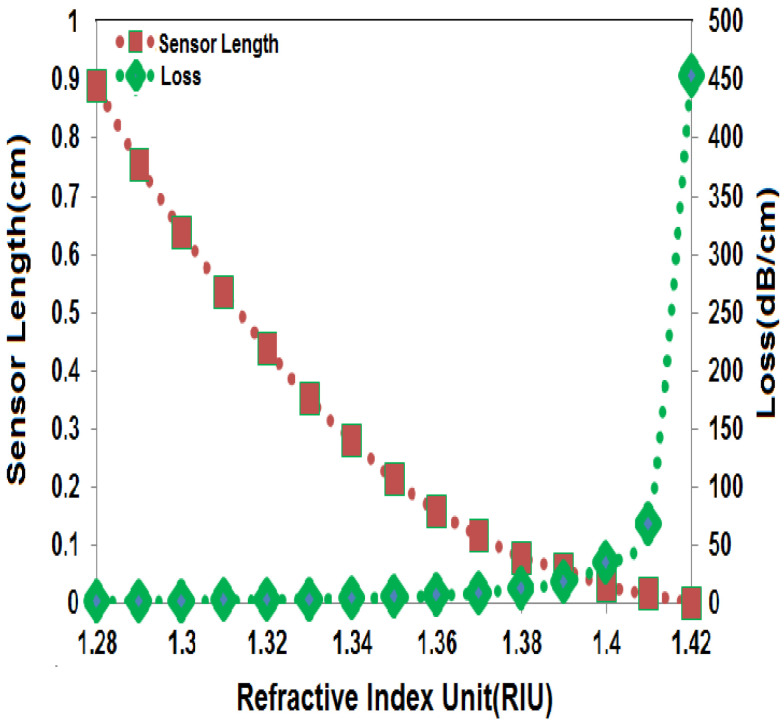
Sensor length and loss vary with different analyte RIs.

As shown in Figure 13, both the sensor length and loss depend on the RI of the analyte. Figure 13 indicates that the analyte with a minimum RI of 1.28 results in the maximum sensor length, whereas the analyte with a maximum RI of 1.42 results in the minimum sensor length. Correspondingly, the analyte with a minimum RI of 1.28 leads to the minimum loss, while the analyte with a maximum RI of 1.42 causes the maximum loss. In general, the sensor length and loss have an inverse relationship in which an increase in sensor length leads to a decrease in loss. 

## 9. Potential Fabrication Methods

The PCF-SPR sensor design proposed in the literature consists of microstructured circular and rectangular air holes, a gold layer, and an analyte layer. The accurate fabrication of the sensor’s geometrical parameters is crucial for achieving optimum sensing performance. Several well-known fabrication methods can be used to fabricate these geometrical parameters, including sol-gel, standard stack-and-draw, stack-and-drilling, 3D printing, extrusion, capillary stacking, injection modeling, and more [74,75]. Sol-gel is a generalized fabrication method that can be used to form basic silica structures. This method involves several steps, including hydrolysis and polycondensation, gelation, aging, drying, densification, and crystallization [76]. The automatic machine-controlled stack-and-draw method can also be used to fabricate circular and rectangular air holes accurately [77]. This method involves using solid, thin-wall, and thick-wall rod tools to create the desired geometries. The fabrication of rectangular air holes in a PCF involves defining a rectangular pattern on a glass rod or preform, drilling the pattern with a laser or mechanical drill, and then drawing the preform into a fiber to elongate and compress the rectangular holes [78]. The resulting PCF has a periodic arrangement of rectangular air holes that can be used to achieve specific optical properties. Thin gold layers can be deposited on the outside of the PCF structure using various methods, including wheel polishing method (WPM), chemical vapor deposition (CVD), automatic layer deposition (ALD), and high-pressure microfluidic chemical deposition methods [79,80,81].

## 10. Promising Applications of PCF-SPR Sensors

The proposed PCF-SPR sensor design has potential applications in various fields due to its good enough sensitivity in detecting the RI of biological analytes within the range of 1.28 to 1.42. This RI range is particularly important as many crucial biochemical solutions and biological analytes have RIs that fall within this range. The suggested PCF-SPR sensor design can be used for real-time monitoring of various solutions and biological analytes, including silicone oil (RI = 1.403), acetone (RI = 1.36), ethanol (RI = 1.361), glucose solution (10%) in water (RI = 1.3477), 50% sugar solution (RI = 1.42), ethylene tetrafluoroethylene (RI = 1.403), human liver (RI = 1.369), white blood cell (RI = 1.36), blood plasma (RI = 1.35), human urine concentration (RI = 1.3415–1.3464), human intestinal mucosa (RI = 1.329–1.338), red blood cell (RI = 1.40), hemoglobin (RI = 1.38), cervical cancer cells (HeLa) (RI = 1.368–1.392), skin cancer cells (Basal) (RI = 1.36–1.38), blood cancer cells (Jurkat) (RI = 1.376–1.390), adrenal gland cancer cells (PC12) (RI = 1.385–1.399), and breast cancer cells (MDA-MB-231 and MCF-7) (RI = 1.387–1.401) [82,83].

## 11. Conclusions

The proposed twin-core, D-shaped PCF-SPR sensor is a promising technology for detection RI of analytes due to its simple structure and high sensitivity. The use of COMSOL Multiphysics software and the finite element method (FEM) allows for the design and optimization of the sensor’s structural parameters, resulting in optimal sensing performance. The proposed sensor can be fabricated using sol-gel, standard stack-and-draw, and automatic layer deposition (ALD) methods, which are widely used in the manufacturing of other PCF-SPR sensors. The results of the simulation show that the PCF-SPR sensor has a high maximum wavelength sensitivity of 9000 (nm/RIU), amplitude sensitivity of 3746 RIU^−1^, FOM of 900 RIU^−1^, and sensor resolution of 1 × 10^−5^ RIU in the x-polarized direction light signal. These promising results suggest that the proposed PCF-SPR sensor could be a potential contender for detecting a wide RI range of biological agents, chemical solutions, and complex diseases in the human body. However, understanding the behaviors of the PCF-SPR sensors in different conditions and applications is crucial to modifying their designs and testing them in the future. Overall, the proposed PCF-SPR sensor could be used for health control, environmental monitoring, effective monitoring of air and water quality, ensuring the safety and quality of food products, and more.

## Figures and Tables

**Table 1:** Performance comparisons between proposed sensor and prior published articles.

Refs.	RI Range	WR (RIU)	FOM (1/RIU)	AS (1/RIU)	WS (nm/RIU)
[64]	NA	5.0 × 10^−5^	NA	574	12,400
[65]	NA	NA	NA	NA	34,600
[66]	1.31–40	8.26 × 10^−6^	NA	1921	12,100
[67]	1.33–1.41	7.767 × 10^−6^	NA	6465	10,300
[68]	1.30–1.44	9.09 × 10^−6^	NA	326	11,000
[69]	1.34–1.38	5.55 × 10^−6^	93.45	2158	20,000
[70]	1.33–1.44	6.94 × 10^−6^	839	1439	63,000
[71]	1.35–1.40	12.5 × 10^−6^	NA	1443	8000
[72]	1.33–1.40	NA	NA	NA	NA
[73]	1.46–1.485	1.22 × 10^−5^	NA	820	23,000
Pro. Sen.	1.28–1.42	1 × 10^−5^	900	3746	9000

**Table 2:** Optimum values of geometrical parameters of the suggested structure.

Area of Air Hole A (µm^2^)	Area of Air Hole B (µm^2^)	Area of Air Hole C (µm^2^)	GLT (nm)	ALT (nm)	PML (nm)
0.0314	1.0	0.6359	26	580	80

## Data Availability

The data are generated using COMSOL Multiphysics software.
